# Declining Performance of Silicone-Based Magnetorheological Elastomers after Accelerated Weathering

**DOI:** 10.3390/ma14216389

**Published:** 2021-10-25

**Authors:** Wibowo Wibowo, Bhre Wangsa Lenggana, Ubaidillah Ubaidillah, Dody Ariawan, Fitrian Imaduddin, Saiful Amri Mazlan, Seung-Bok Choi

**Affiliations:** 1 Mechanical Engineering Department, Faculty of Engineering, Universitas Sebelas Maret, Surakarta 57126, Indonesia; wibowo69@staff.uns.ac.id (W.W.); bhrewangsa20@gmail.com (B.W.L.); dodyariawan@staff.uns.ac.id (D.A.); fitrian@staff.uns.ac.id (F.I.); 2 Engineering Materials and Structures (eMast) iKohza, Malaysia-Japan International Institute of Technology (MJIIT), Universiti Teknologi Malaysia, Jalan Sultan Yahya Petra, Kuala Lumpur 54100, Malaysia; 3 Department of Mechanical Engineering, The State University of New York, Korea (SUNY Korea), Incheon 21985, Korea; 4 Department of Mechanical Engineering, Industrial University of Ho Chi Minh City, 12 Nguyen Van Bao Street, Vap District, Ho Chi Minh City 70000, Vietnam

**Keywords:** magnetorheological, elastomer, magnetorheological elastomer, MRE, weather, accelerated, rubber, composite, rheological

## Abstract

Magnetorheological elastomers (MRE)-based products are usually located in an area directly exposed to sunlight and rain. However, there is no specific research on the behavior of MRE after accelerated weathering. Therefore, in this study, the changes to the chemical and rheological properties of both isotropic and anisotropic MRE after accelerated weathering were examined. Treated and untreated specimens were compared. MRE specimens with 40% by weight CIP were prepared with no current excitation and another sample was prepared with 1.5 T of magnetic flux density. Each specimen was treated in an accelerated weathering machine, Q-Sun Xe-1 Xenon Test Chamber, under a UV light exposure cycle and water spray. A material characterization was carried out using FTIR and a rheometer to determine the changes to the chemical and rheological properties. The morphological analysis results showed that after the weather treatment, the surface was rough and more cavities occurred. The rheometer test results showed a significant decrease in the storage modulus of each treated MRE specimen, unlike the untreated MRE specimens. The decrease in the storage modulus value with currents of 0, 1, 2, and 3 Amperes was 66.67%, 78.9%, 85.2%, and 80.5%, respectively. Meanwhile, FTIR testing showed a change in the wave peak between the untreated and treated MRE specimens. Thermogravimetric analysis (TGA) also showed a decrease in MRE weight for each specimen. However, for both treated and untreated MRE specimens, the decrease in TGA was not significantly different. In all the tests carried out on the MRE samples, weather acceleration treatment caused significant changes. This is an important consideration for developers who choose silicone as the MRE matrix.

## 1. Introduction

The development of rubber products has progressed rapidly. One of these developments is engineered rubber products, widely applied in various engineering fields. These products have excellent physical properties and durability, as well as the ability to deform. Rubber composites are one type of engineered rubber product widely applied in various fields such as marine [1], structural, automotive [2,3], and aerospace [4]. Several studies have shown that rubber composite products have greater environmental sensitivity compared to metal structures, especially in their application in the space sector. However, engineered rubber products, such as composites, are highly susceptible to the conditions of ambient temperature, humidity, heat, and chemicals they encounter. In general, the aging of rubber composites is caused by chemicals [5,6], thermal effects, heat [7,8], oxidative destruction [9,10,11], and moisture [12]. Thus, the weather conditions in which the applications of rubber composite products occur can negatively affect their physical properties [13]. A particular issue is the degradation or aging stability of rubber composites. The aging phenomenon is an irreversible process that can significantly change their physical, mechanical, and rheological properties [13,14]. The change in interface adhesion in reinforcement and rubber causes hardening of the rubber composite, resulting in brittleness and a reduced lifetime. 

In addition to general rubber products applied in the automotive sector, such as tires or other specialty rubber products, rubber composites in the form of sensors and magnetic and electromagnetic field protection materials have been proposed in recent decades. For this kind of usage, a new type of rubber composite with a nonconventional filler as an integrated active component is required [15]. One way to achieve this purpose is to prepare a new material with a specific filler such as magnetic particles. Magnetorheological elastomers (MRE) are a new generation of materials widely explored for their excellent flexibility, ease of formation, and sensitivity to magnetic fields [15,16]. However, despite the capabilities of MRE as a composite material, it remains highly vulnerable to damage due to the influence of the surrounding environment [17]. MRE aging affects the mechanical properties and durability of MRE, resulting in damage. One example is the study conducted by Kruzelak et al. [10], which showed an increase in the crosslink density of magnetic composites due to the formation of rubber chain oxidation during oxidative aging. They observed that the mechanical properties associated with the rubber magnetic composites’ modulus and hardness increased compared to the samples without aging. Masbowski et al studied thermo-oxidative aging in MRE samples. [18] MRE samples with carbonyl iron particles (CIP) and carbon black fillers were treated by being placed in a drying oven at 70 °C for two weeks [19]. The mechanical properties of the MRE slightly deteriorated due to the oxidative aging process. Other researchers drew similar conclusions; tensile strength, tear strength, and hardness decreased significantly up to 72% with the increase in temperature and aging time. This was due to the molecular configuration changes accompanied by hydrothermal deterioration that caused microgaps to develop between the matrix and the fill/matrix interface.

Treatment related to MRE degradation has been conducted in several forms, such as thermal treatment, but some of these treatments could not represent weather behavior. Based on previous research, rubber composite/MRE studies have been limited to specific rubber matrices and mainly focused on mechanical properties and crosslinking [20]. One study involved the use of RTV silicone rubber as a massive MRE matrix because of the ease of fabrication. However, intensive studies on the resistance of MRE based on RTV silicone to natural conditions such as sunlight, heat, and rainfall have not received attention [21,22]. Meanwhile, the application of rubber composite products such as MRE has been mainly in environments directly exposed to sunlight and rain [23,24]. Therefore, this article contributes to understanding the effect of weathering by using an accelerated weathering machine on MRE specimens based on silicone RTV, both isotropic and anisotropic types. New findings were realized by examining the rheological properties and material deterioration under infrared testing. The main finding could be important for researchers or designers in deciding whether to use silicone as the MRE matrix.

## 2. Materials and Methods

### 2.1. Samples Preparation

**Figure 1:**
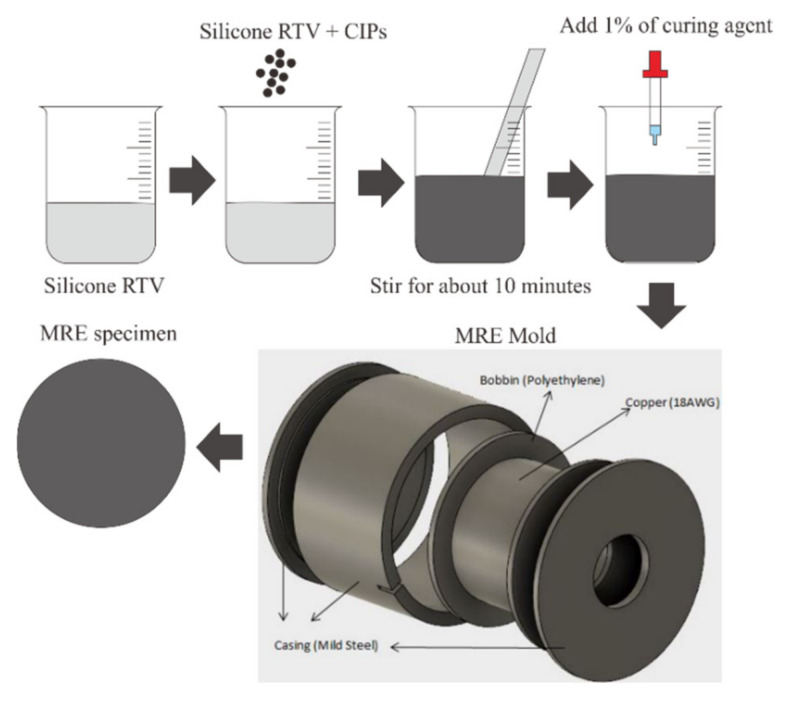
MRE sample preparation.

In this study, MRE samples were prepared based on silicone RTV consisting of 40 wt.% CIPs and silicone RTV as a suspending medium. The preparation of the MRS samples is shown in Figure 1.

Both isotropic and anisotropic types were made using the same equipment. For the anisotropic MRE samples, the mixed silicone RTV and CIP were cured under the influence of a magnetic flux density of 0.5 T. This flux magnetic value was obtained by applying 1 ADC current. To determine the value, the flux density was measured using a gauss meter before the mold was filled with the mixture. The MRE sample was stirred conventionally for about 10 min at room temperature (25 °C) until it was visually homogenous. Then, a 1% curing agent, NS 625 B (Nippon Steel) was added to the stirred MRE sample before it was poured into the mold. To remove small bubbles, the mixtures were placed in a vacuum chamber for degassing purposes [15].

### 2.2. Sample Characterization

The test method followed the standard test method for rubber deterioration using an artificial weathering apparatus. This process was conducted in an accelerated weathering machine Q-SUN Xe-1 Xenon Test Chamber and ASTM D750. The Q-SUN Xe-1 Xenon Test Chamber is a tabletop chamber that reproduces the damage caused by full-spectrum UV light using a single xenon arc lamp with optional water spray and chiller which is manufactured at Q-Lab Florida, FL, USA.

As stated in ASTM D750, the specimens should have a maximum thickness of 0.75 mm and a minimum thickness of 0.60 mm (0.025 in); however, the width of the specimens is not critical. Along with the ASTM standard thickness, samples with a thickness of 1 mm were also treated based on the needs of the rheology testing, which requires a minimum thickness of 1 mm. The default exposure was 102 min light only, followed by 18 min light plus either water spray on the front surface or immersion in water. The black panel temperature (BPT) for this test was 63 °C during the dry period of exposure to light, with a relative humidity of 60% during this exposure. The irradiance level was controlled at 340 nm. This test ran an 8 h cycle of UV exposure in an uninsulated black panel with a temperature of 60 °C, followed by a condensation cycle of 4 h in the dark, with wetting and an uninsulated black panel temperature of 50 °C. The samples obtained were MRE samples with and without treatment, for both isotropic and anisotropic types. 

The prepared samples were then characterized by several tests such as morphology, rheology, and Fourier transform infrared (FTIR). To examine the surface of the treated and untreated samples, image capture for micrograph analysis was performed at ten magnification (M10) using a Euromex microscope model “F” range, Holland by Euromex Microscopen BV, Arnhem, Netherlands. This enlargement was clear enough to identify differences between the treated and untreated samples. Micrograph analysis was carried out on the surface of the samples, since during the treatment with an accelerated weathering machine, the sample surfaces were directly exposed to both UV rays and water. The MRE samples for rheometer testing were cut to a diameter of 20 mm. This test was performed using a Rheometer MCR 302 Anton Paar Companies machine. The amount of the MRE sample between the CIPs and silicone rubber medium in this study was based on the weight ratio. The two ingredients were stirred manually at room temperature for 10 min until homogeneous. After homogeneity was reached, each sample was poured on the mold and levelled before clotting. The samples were tested with a rheometer at 25 °C. In addition to rheometer testing, FTIR testing was carried out on samples to determine any changes or differences in chemical compounds between the untreated and treated MRE samples. The MRE samples to be tested by FTIR were crushed until smooth using a mortar stamper. FTIR testing used a Shimadzu Prestige 21 IR, Japan, with a wavelength specification of 12500–240 cm^−1^ and an accuracy level of ±0.125 cm^−1^. The spectrum resolution on this machine ranged from 0.5 to 2 cm^−1^.

## 3. Results and Discussion

### 3.1. Micrograph Analysis

**Figure 2:**
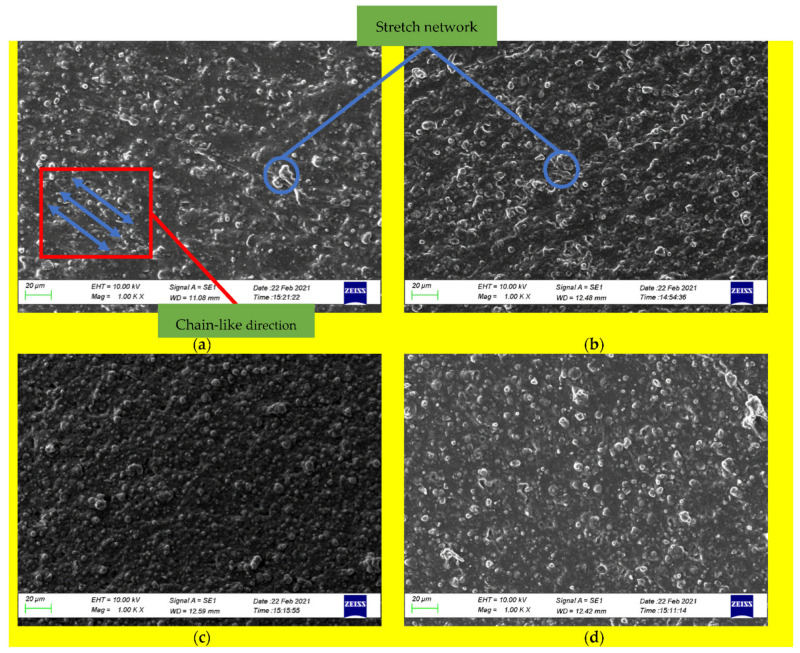
SEM results of cross-sectional MRE sample slices; (**a**) anisotropic untreated; (**b**) anisotropic treated; (**c**) isotropic untreated; (**d**) isotropic treated.

Micrograph studies of the surfaces of the treated and untreated MRE samples were essential to analyze the degree of degradation. Figure 2a shows an untreated micrograph sample, and Figure 2b shows a micrograph sample with weather treatment using an accelerated weathering machine. The effects of surface degradation are evident in these images [25,26], with the results showing a clear difference.

After treatment, the MRE sample surface was more irregular than that of the virgin sample. The change in the roughness of the treated samples was clear, even using a regular microscope. Surface defects were possibly due to the evolution of low molecular degradation products, which allow the penetration of water and possible decomposing agents (e.g., enzymes) into the bulk of the polymer and facilitate further environmental degradation. This phenomenon was also found by other researchers who tested MRE with thermal aging [27].

Micrograph analysis was performed using an SEM (Scanning Electron Microscope) ZEISS EVO 10 by Carl Zeiss Company, Jena, Germany. The analysis was carried out with a magnification of 1000 times for both sample variations. SEM testing in this study aimed to see a clearer description of the elastomeric structure with a larger magnification scale, so that a comparison could be completed between the MRE samples with and without weather treatment. The test was carried out on the surface of the sample and the section of the sample slice, so that the chain structure formed on the MRE could be seen. The samples compared were isotropic and anisotropic MRE samples, treated and not weather treated. The results are shown in Figure 2a,b; the anisotropic MRE sample without weather treatment had a more neatly arranged structure and its appearance was a chain arrangement line that was cured with 1 A input. Weather-treated anisotropic MRE samples showed a more irregular (random) structure. A stretch network was seen more in the MRE samples with weather treatment, due to the samples being exposed to UV light alternately and sprayed with water continuously during the treatment process. The existence of the stretch network and the random arrangement of the chains caused the rheological properties of MRE to decrease significantly, as evidenced by the graph of the rheological test results.

For the MRE sample without weather treatment, the chain link structure of the sample slices was still clearly formed; while in the weather treated sample, the path was not visible because it appeared more random. This caused a decrease in the properties in the sample, according to the results of the graph. The weather treatment was carried out only on the surface of the sample, but the impact that occurred to the center of the sample was shown through the slices of the sample being tested. The results of the SEM test are shown in Figure 2. Data were collected using the same method and magnification. In addition, the number and size of stretch networks on the surface were also significantly different. At the same magnification, the surface of the anisotropic MRE samples with weather treatment was faded and grayer. This is, of course, a result of the UV rays being alternately exposed to the sample with constant water spray. In terms of color changes between the surface and the middle/inside of the MRE samples analyzed by SEM, the interior tended to remain the same color with or without treatment, since the inside was not exposed to the UV light. Meanwhile, judging from the number and size of the resulting stretch networks, the MRE samples with the weather treatment were more numerous and evenly distributed over all parts of the surface with a smaller size than the untreated MRE samples. However, in both of them, the chain structure path was not visible as clearly seen in the sliced MRE samples. The structure of both were irregular. 

**Figure 3:**
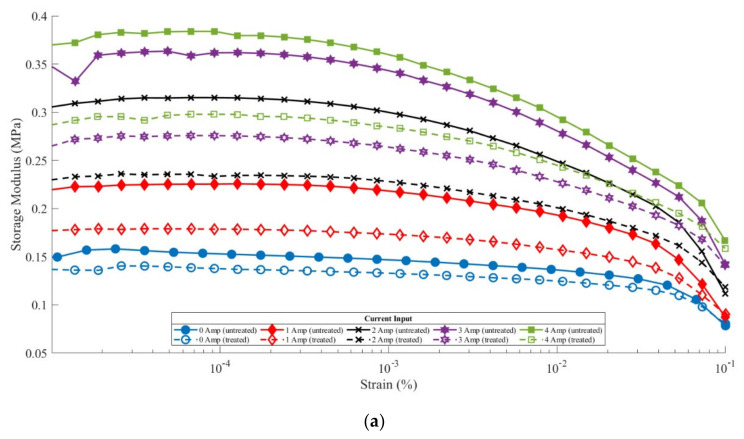

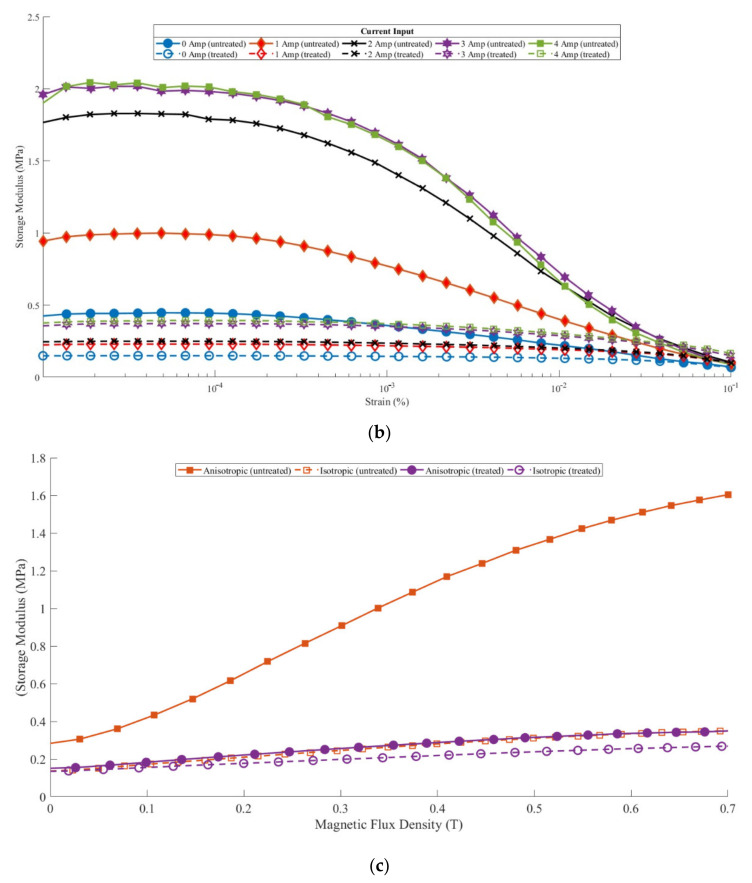
Results of rheometer characterization (**a**) isotropic; (**b**) anisotropic; and (**c**) comparison of anisotropic and isotropic under ramp frequency test.

Isotropic MRE samples were also tested with the same method and magnification. Overall, the SEM results showed similarities, as presented in Figure 2c,d. The samples with the weathering treatment had more bubbles. This occurred in all the tests, both on the surface and on the slices. However, in contrast to the anisotropic MRE sample where a chain-like structure was formed, in this sample, structural bonds such as chains were not formed, because the manufacturing process did not use a magnetic field. This is shown in Figure 2c,d, with the 1000 times magnified samples of MRE slices showing a very random distribution of bonds. The difference in the number and size of bubbles in this isotropic MRE sample was not significant compared with the previous anisotropic sample. This is evidenced by the decrease in storage modulus, which is not significant, as shown in Figure 3c. The results are different from the decrease that occured in the anisotropic MRE samples.

### 3.2. Rheological Properties

The storage modulus of all samples with and without weather treatment is depicted in Figure 3. As shown in Figure 3, the storage modulus at each MRE sample flow strength, both anisotropic and isotropic, decreased. In Figure 3c, the treated anisotropic MRE sample showed a storage modulus value that was almost identical to the storage modulus of an isotropic MRE sample without treatment. This indicates that weather treatment can damage the particle chain arrangement that occurs in anisotropic MRE samples [28,29,30,31]. The breakdown of the particle chain arrangement occurred in all anisotropic MRE specimens and brought all storage modulus values close to the storage modulus of the isotropic MRE samples.

Figure 3 shows that the anisotropic MRE sample without treatment had a peak storage modulus value of 1.9 MPa at the current input of 4 A. Simultaneously, the peak storage modulus value of the anisotropic MRE sample treated at the same current input (4 A) was 0.37 MPa.

**Figure 4:**
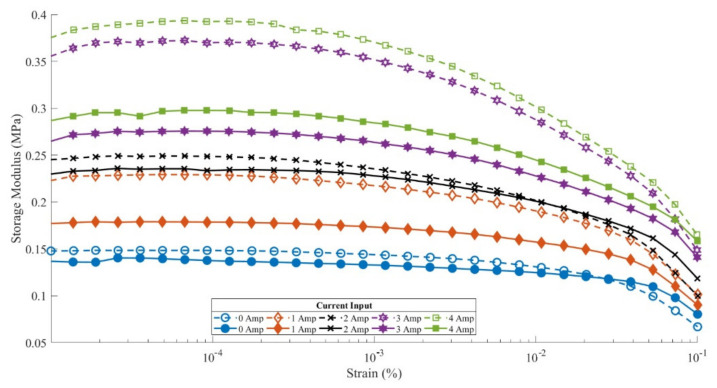
Results of MRE sample rheometer test for anisotropic treated and isotropic untreated.

This shows that the storage modulus value decreased by 80.5% due to the influence of weather (exposure to UV rays and water spraying), which was carried out in a test cycle. A decrease in the storage modulus value occurred in all current inputs in the rheometer test. The decrease in the storage modulus value with currents of 0, 1, 2, and 3 Amperes was 66.67%, 78.9%, 85.2%, and 80.5%, respectively. This reduction was highly significant compared to that of the isotropic MRE sample, as shown in Figure 4. The anisotropic MRE sample had a high storage modulus value due to the arrangement of the particle chains formed with the input of an electric current during the curing process of creating the samples.

A ramp frequency or frequency sweep is a particularly useful test as it enables the viscoelastic properties of a sample to be determined as a function of timescale. The storage modulus can be used as a measure of the elastic component of the sample and similarly, the loss modulus–the viscous component of the sample. Whichever modulus is dominant at a particular frequency will indicate whether the fully structured material appears elastic or viscous, in a process on a similar time scale. The mechanical response of most dispersion coatings is viscoelastic, since the presence of suspended solids, high additive concentrations, colloidal thickeners, etc., will induce a structure upon the bulk phase.

Figure 4 shows the results of the isotropic MRE sample rheometer test on the treated and untreated MREs. The peak value of the storage modulus of the isotropic MRE without treatment only reached 0.37 MPa at 4 A current input when tested. This value was almost the same as the storage modulus value of the anisotropic MRE treated sample. Meanwhile, the isotropic treated MRE sample’s storage modulus value only reached a peak value of 0.28 MPa. This shows that the decrease in the value of the isotropic MRE sample’s storage modulus at the 4 A current input was 24.3%. This reduction value is not comparable to that of the anisotropic MRE sample, which achieved a mean reduction of up to 78.3%. This occurred because the arrangement of the particle chains in the isotropic MRE sample was irregular, so the weather treatment given to the sample did not damage the arrangement but only degraded its rheological properties [32,33].

### 3.3. FTIR Testing

**Figure 5:**
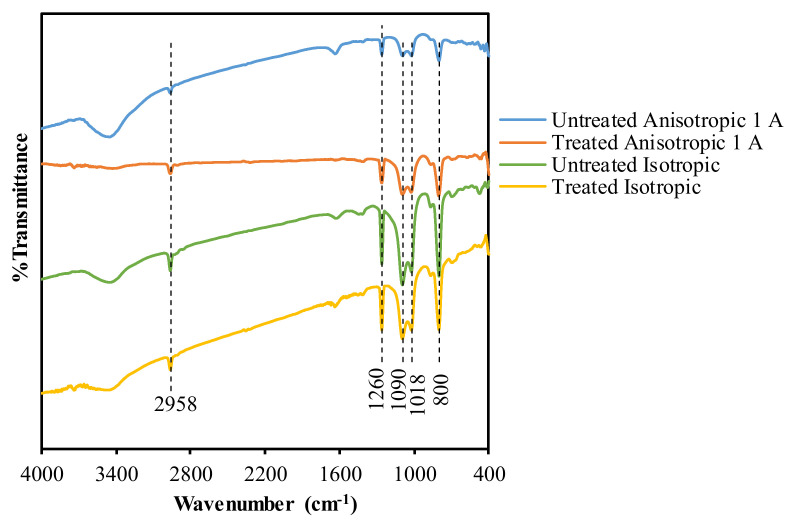
FTIR results.

FTIR testing carried out on the test samples produced data in the form of a graph of wavelength (cm^−1^) vs. % transmission, as shown in Figure 5. The graph shows the existence of a transmission in the form of a peak to be interpreted. This interpretation shows the bonds formed in the test specimens after weathering treatment and in the specimens without treatment.

The functional groups of the sample were elucidated with Fourier Transformation Infrared (FTIR) spectroscopy and the result is presented in Figure 5. The peak at around 2958 and 1260 cm^−1^ was attributed to the stretching and bending vibration of Si–(CH_3_)_2_ [34]. Meanwhile, two absorption bands at 1090 and 1018 cm^−1^ were assigned to the characteristics of silicone rubber for –Si–CH_2_–CH_3_ [35]. Meanwhile, the peak around 800 cm^−1^ corresponded to silanol groups that could be deconvoluted to three peaks, as illustrated in Figure 7, using Equation (1). The peaks at 795, 800, and 810 were Si–O–Si, terminal Si–OH and unbonded Si–O, respectively [36]. FTIR deconvolution is a method of estimating quantitative interaction in a certain functional group [37]. The intensity of the bond formation in each specimen appeared to be the same. However, there was a difference at a wavelength of 3451 cm^−1^, as the untreated specimens showed the formation of a peak with a reasonably comprehensive intensity, compared to the treated specimens, both anisotropic and isotropic. There was almost no peak appearance in the treated specimens, as shown in the results of the treated anisotropic specimens. This indicates that the OH group formed before the treated specimen faded due to the influence of UV exposure.

**Figure 6:**
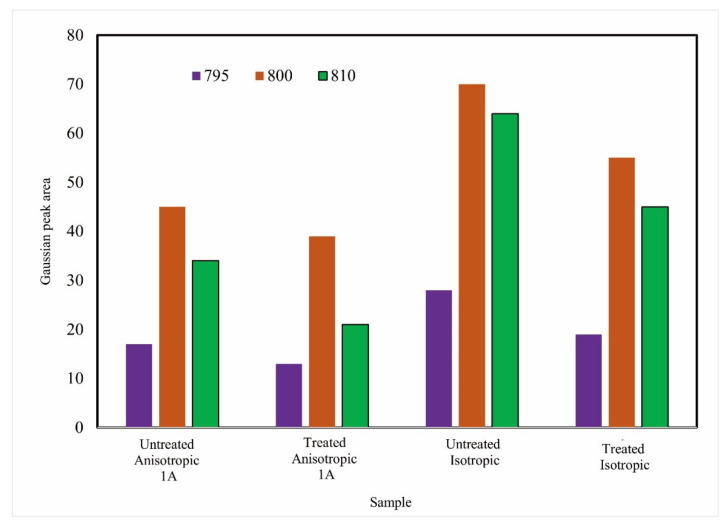
Gaussian peak area at 800 cm^−1^.

**Figure 7:**
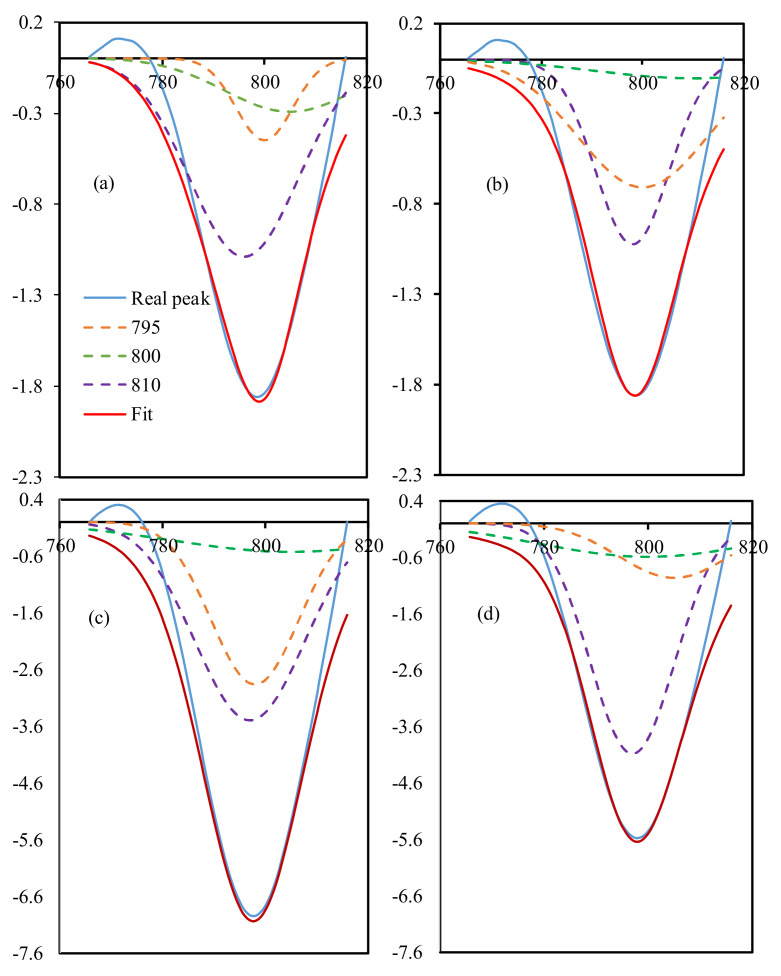
FTIR deconvolution of peak 800 cm^−1^.

Of all the tested MRE specimens, no absorption was found at 560–570 cm^−1^, which should be the iron oxide or Fe–O absorption region [38,39]. This behavior is called a Binder bond [40]. The binder bond occurs due to the neutralization of the carbonyl charge so that it cannot interact with infrared wavelengths. Meanwhile, using the deconvolution method, the Gaussian peak area is presented in Figure 6. The peak area of the treated sample showed decreasing trends, possibly due to the iron filler detaching from the silicone rubber. The Figure 7a–d show the consistency of the deconvolution results at the three peaks. Finally, the iron carbonyl as a filler had no chemical interaction with the silicone rubber because the rubber coated the iron filler, as reported in other research [41].

(1) $$Y=\frac{A}{w}\sqrt{\frac{4\ln2}{\pi }}\frac{1}{4\ln2}\frac{{\left(x-xc\right)}^{2}}{w^{2}}$$

where *A* and *w* are the area and full width of the graph, respectively, with *xc* as the center.

### 3.4. Thermogravimetric Analysis (TGA)

**Figure 8:**
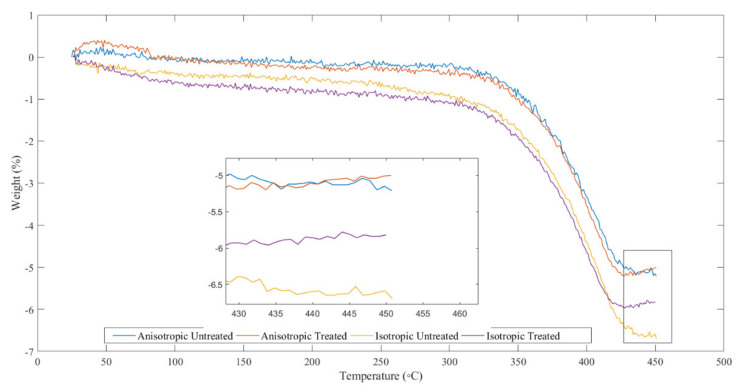
Thermogravimetric curves of MRE specimens.

Thermogravimetric analysis was performed using a TGA Q50 by TA Instruments, New Castle, DE 19720, USA. The magnetorheological elastomers test sample had a mass of about 5–10 mg with a temperature variation of 30 to 600 °C used. The test was carried out using a heating rate of 20 °C/min under a nitrogen gas environment (flow rate 100 mL/min). An expansion probe was equipped to measure the change in dimensions of the samples measuring 5 × 5 × 10 mm. Data were collected at a heating rate of 10 °C/min from an ambient temperature of 150 °C while the initial load was set at 0.05 N. Figure 8 shows the thermogravimetric results of several variations of sampled magnetorheological elastomers. The tests were carried out on the isotropic and anisotropic samples and the results were also compared between samples with weather treatment and without.

Boczkowska et al. (2006) carried out a similar analysis using MRE samples. Thermogravimetric testing is one part of thermal analysis, which determines the level of stability of a material against external temperatures. In addition, the oxidative mass reduction, and its decomposition performance can also be determined through this analysis. Boczkowska et al. (2006) reported that at a temperature of around 600 °C additional iron particles in the elastomeric matrix will increase stability [42], indicated by the presence of lower oxidative mass loss. The thermal conductivity of the MRE sample was said to be improved due to the higher magnetic particles in the MRE sample, which increased its thermal conductivity. This happens because the distance between the particles is closer. Therefore, the flow of heat energy distributed throughout the sample will be greater if the MRE element has a higher number of iron particles. 

In this study, the level of thermal stability of the MRE sample was evaluated using thermogravimetric analysis (TGA) Q50 by TA Instruments, New Castle, DE 19720, USA. In addition, the content of carbon black and ash compounds in the rubber used in the sample can be observed with this analysis. Figure 8 shows the TGA thermogram of the MRE samples. The rubber content of all samples completely decomposed. The initial temperature of the volatile compounds released in all samples was greater than 300 °C. The volatile content of the samples was determined to be around 5.15–9.85%. There was a mass loss of five percent (T5), increasing from 297.65 °C to 421.65 °C (anisotropic MRE with weather treatment). Mass loss in other samples, anisotropic MRE without treatment and isotropic with and without treatment, also increased at the same temperature. The last curve was maintained at the same weight until the next transformation. The content of carbon and CI particles changed at a temperature of more than 450 °C. The weight reduction reflects the composition of the CI particles in the rubber and confirms the substance of the rubber. Lower CI particle content indicates a higher sample weight reduction. In this study, the weight loss of each sample at the end was 4.79, 4.83, 3.35, and 4.03% for the anisotropic MRE samples without treatment, with treatment, isotropic without treatment, and with treatment, respectively. The remaining elements (at 450 °C) as described in Table 1 were carbon black and CI particles. The test results showed that the thermal stability of the anisotropic MRE samples was greater than the isotropic MRE samples. This relates to the chain-like formation of particles in the anisotropic MRE samples. The residues of the MRE samples after the thermogravimetric test are shown in Table 1 below.

The tested MRE samples showed that the residue from the MRE samples with both isotropic and anisotropic weather acceleration treatments was greater than the samples without treatment. Wang et al. found a similar pattern with different compositions of carbonyl iron particles in the MRE content and concluded that the greater thermal stability was shown by the MRE material, which had a higher carbonyl iron particle content [43,44]. The residual results shown in Table 1 are comparable. The residue produced by the MRE sample without weather treatment showed a significant difference from the treated sample, because the carbonyl iron particle content in the treated MRE samples had decreased or even been damaged, as previously described in the micrograph analysis, where many cracks and discoloration were visible. The rheological results were also in accordance with the statement that accelerating weather treatment causes a decrease in the performance of the material.

## 4. Conclusions

Weather treated and untreated MRE samples were characterized. The results from the analysis micrograph showed a significant difference between the treated and untreated samples. The degradation of the treated samples was evident in their increased roughness and the particle arrangement’s surface irregularity. The results of the characterization test using rheology showed a highly significant reduction in the storage modulus. This decrease was up to 80.5% and occurred due to the aging of the samples treated using an accelerated weathering machine with exposure to UV light and continuous and repeated spraying of water on the sample. This process occurred due to the breaking of the chain of particle bonds arranged in the MRE sample; hence, the MRE particle chain formed in the anisotropic sample was damaged and resembled an isotropic sample. In FTIR testing, each sample showed little difference, with the appearance of a peak at the same wavelength with almost the same intensity. Nevertheless, a distinguishing factor was that at a wavelength of 3451 cm^−1^, the intensity of the untreated samples in forming OH groups was very low. Even the untreated anisotropic sample barely formed a peak at that wavelength. Thus, based on the results of the MRE characterization after weather treatment, the aging of the samples was faster, so a decrease in sample performance occurred. Exposure to UV light and water spray greatly affected the performance of the MREs, which are widely applied in open environments. This decrease in performance was evident in the rheological characterization test, which showed that the storage modulus of the treated sample decreased significantly compared to the untreated samples. This represents a new challenge for ongoing research in the field of advanced materials and magnetorheological elastomers, in particular.

## Figures and Tables

**Table 1:** Residual mass of MRE specimens.

Sample	Residue (%)
Anisotropic without treatment	1.95
Anisotropic with treatment	2.25
Isotropic without treatment	0.05
Isotropic with treatment	0.6

## Data Availability

Data sharing is not applicable for this article.
